# Editorial: Structure and Function of the Insula-Claustrum Region

**DOI:** 10.3389/fnana.2020.00014

**Published:** 2020-04-08

**Authors:** David Reser, Fabienne Picard

**Affiliations:** ^1^Department of Physiology, Biomedicine Research Institute, Monash University, Melbourne, VIC, Australia; ^2^Monash Rural Health Churchill, Churchill, VIC, Australia; ^3^University Hospitals of Geneva and Faculty of Medicine, Geneva, Switzerland

**Keywords:** claustrum, insula, cortex, mammal, special collections

We are pleased to present this collection summarizing the current state of the art and ongoing progress into understanding the complex anatomy, physiology, and functional characteristics of the mammalian insula-claustrum region. The impetus for this special topic comes from the wide array of functions ascribed to this area and its broad connectivity to other cortical regions. Cortical connectomic studies from an array of mammal species have shown that both the insula and claustrum display patterns of connections which suggest that they feed into multiple networks, and may influence almost every aspect of brain function. Recent advances have also helped to resolve the developmental origin and relationships of the claustrum complex (Binks et al.; Watson and Puelles, 2017; reviewed in Smith et al., 2019). Though some discussion persists, clarification of the embryological claustrum plan will facilitate identification of homologs of this structure in non-mammalian species (Wulliman, 2017). Despite this richness, the anatomy and physiology of the insula, and especially the claustrum, are often taught as an afterthought in Neurology and graduate-level Neuroscience programs, and our aim with this collection is to provide a comprehensive, accessible resource for investigators whose data have led them to the insula-claustrum area, as well as for student and early-career researchers who may be looking for interesting targets of study.

Evrard provides a fresh and detailed perspective on the anatomy and function of the primate insula, including a review of the current understanding of Von Economo neurons (VEN) in the primate, and how they may relate to VEN characterized in other species. He also presents a functional-spatial model of insular cortex organization which includes its putative roles in sensorimotor and cognitive-emotional processing.

Bossaerts presents a new and original theory of learning that he calls “reference-model based learning,” in which the goal is “to bring outcomes in line with expectations in the reference model.” In this theory, the agent does not attempt to optimize but to satisfy. This theory allows the agent to be happy without always having to optimize. The agent will feel satisfied if actions do not lead to excessive surprises.

Qadir et al. explore the connections of the anterior insula with the claustrum and anterior cingulate cortex (ACC) of the mouse through adeno-associated virus neuronal tract tracer injections. Their study supports that the claustrum is involved in salient stimuli detection through a connection with the ACC. They show a weak projection of the claustrum to the anterior insula and confirm labeling of the claustrum via viral injection into the ACC.

Exploring the resting state connectivity of the rat claustrum using high-field strength imaging, Krimmel et al., confirm that the well-known structural connection between cingulate cortex and claustrum has a resting state functional counterpart, which is distinct from the resting state connectivity of surrounding structures. Resting-state functional connectivity of the claustrum will be an important measure for testing hypotheses of claustrum function, as it is the most common method for identification of cortical networks, including default mode, task positive, and salience networks. Moreover, the analytical method employed by Krimmel et al., small region confound correction (SRCC), holds promise for studies of other small, but highly interconnected subcortical areas, including, e.g., ventral pallidum, habenula, and intralaminar nuclei of the thalamus.

Pham et al. examine the myeloarchitecture and chemoarchitectonic features of the marmoset claustrum to clarify the internal structure of the claustrum complex, and specifically to better understand the relationship of the so-called “insular” claustrum and the closely associated dorsal endopiriform nucleus. They identify several subdivisions of the dorsal endopiriform by myeloarchitectural features, with at least one subdivision, the dorsal division of the dorsal endopiriform, exhibiting distinctly different connectivity than the insular claustrum, as assessed by diffusion weighted imaging.

The always difficult, but essential, challenge of attempting to understand the homology of brain structures in phylogenetically distant species was addressed by Binks et al., who examined the topology of pallial structures, including the claustrum, in rodents and primates. Their study corrects several persistent errors of interpretation in claustrum morphology, and establish both genetic/developmental, and spatial compartmentalization of the claustrum complex. They convincingly demonstrate both the lateral pallium origin of the classical dorsal claustrum, and largely confirm the segregation of the claustrum complex into at least two divisions, each with a distinct developmental character. They also note the lateral pallial origin of the cells destined to form insular cortex, which are adjacent to the dorsal “insular” claustrum, but separate to the cell population giving rise to the dorsal endopiriform nucleus, which originates in the ventral pallium. This revised plan will serve to standardize the nomenclature and boundary identification of the respective subdivisions- a crucial foundation for interpreting future cross-species comparisons.

The final two papers in this collection describe important advances for translation of claustrum-insula studies into the domain of human brain function, health, and disease. Kurada et al. examine claustrum involvement in consciousness-altering seizures, using the rat kainic acid (KA) kindling model. Using evoked cortical potentials driven by electrical stimulation of the claustrum, this group had previously shown that performance on an operant task decreased in proportion to the intensity of claustrum stimulation. That finding is followed up here with important control experiments in which stimulation of surrounding and adjacent extra-claustral regions does not yield a similar impact on the operant task. Moreover, they describe the temporal pattern of claustrum involvement in KA-induced seizures, and show that ictal activity in the claustrum precedes other structures, including the hippocampus. In context with other recent data indicating a role for the claustrum in seizure disorders, this finding represents a novel and potentially quite fruitful line of inquiry for experimental clinical Neurology.

Finally, interpretation of animal studies in the context of the structure and function of the insula-claustrum region in the human brain has been made easier by the work of Ibrahim et al., who undertook a transcriptomic survey of adult and neonatal human brains to identify enriched markers in the insular-claustrum region. Among the key findings of this survey are markers which further implicate the claustrum and insula regions in seizure disorders and disorders of intellectual development. Importantly, Ibrahim et al. have identified several genes which show distinct patterns of expression between the claustrum and insula, and suggest potential functional differences in these structures. Resolution of the activity in the claustrum complex from insular cortex is difficult in functional imaging studies, due to the proximity and commonality of blood supply between the two structures, so identification of molecular markers which may differentiate them is in itself a significant advance. Furthermore, the range of developmental, behavioral, and signaling functions encompassed by the genes showing high expression in the insula-claustrum region is consistent with the degree of connectivity exhibited by these structures, and with their presumed importance in a broad array of critical autonomic and extrinsic sensory functions, motor outputs, and states of awareness.

## Author Contributions

FP and DR contributed equally to creation and editing of this work.

### Conflict of Interest

The authors declare that the research was conducted in the absence of any commercial or financial relationships that could be construed as a potential conflict of interest.
